# Molecular Basis of Stationary Phase Survival and Applications

**DOI:** 10.3389/fmicb.2017.02000

**Published:** 2017-10-16

**Authors:** Jananee Jaishankar, Preeti Srivastava

**Affiliations:** Department of Biochemical Engineering and Biotechnology, Indian Institute of Technology, New Delhi, India

**Keywords:** stationary phase promoters, stationary phase gene expression, plasmid vectors, sigma factor, stationary phase

## Abstract

Stationary phase is the stage when growth ceases but cells remain metabolically active. Several physical and molecular changes take place during this stage that makes them interesting to explore. The characteristic proteins synthesized in the stationary phase are indispensable as they confer viability to the bacteria. Detailed knowledge of these proteins and the genes synthesizing them is required to understand the survival in such nutrient deprived conditions. The promoters, which drive the expression of these genes, are called stationary phase promoters. These promoters exhibit increased activity in the stationary phase and less or no activity in the exponential phase. The vectors constructed based on these promoters are ideal for large-scale protein production due to the absence of any external inducers. A number of recombinant protein production systems have been developed using these promoters. This review describes the stationary phase survival of bacteria, the promoters involved, their importance, regulation, and applications.

## Introduction

The majority of the microorganisms around us in air, sea water, and soil are predominantly present in stationary phase (Gefen et al., 2014). The natural habitat of microorganisms often contains limited nutrients due to which rapid growth is usually hampered. Apart from nutrient deprivation, there are other conditions, including physical and chemical stresses, which result in unbalanced growth. All these events result in many changes at the molecular level. These molecular changes are comparable to those observed during the stationary phase of bacteria as witnessed in laboratory studies. The entry of bacteria to the stationary phase can be caused by different factors, including limitation of a specific essential nutrient, accumulation of toxic by-products, presence of stress factors such as changes in pH, temperature, osmolarity, etc. As the cell enters this phase, there is a reduction in cell size and the DNA/protein ratio is said to increase during transition to stationary phase (Nystrom, 2004). The stationary phase has received much attention due to the pattern of protein synthesis in this phase and also because of survival strategies adopted by bacteria. Numerous physiological, morphological, and gene expression changes are observed when a growing cell enters the stationary phase. These are discussed in the following sections.

## Physiology of the Stationary Phase

In the stationary phase, the cells become spherical and smaller with a rigid cell envelope, the cell wall is highly cross-linked, membrane fluidity reduces, and cells activate the stringent response mechanism in order to survive the calamity. The activation of this mechanism allows the bacteria to reprogram the gene expression pattern to adapt to different stresses. Two key components of the bacterial stringent response are ppGpp and pppGpp (which are explained in a later section). As a consequence, the cells divert their resources away from growth toward synthesizing amino acids so as to promote survival till nutrient conditions improve.

**FIGURE 1:**
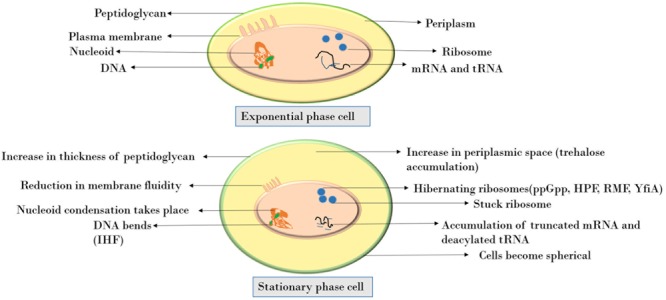
Comparison of molecular and cellular changes in exponential vs stationary phase.

**Figure 1** depicts the various changes observed in a cell when it enters the stationary phase. The peptidoglycan layer, being the stress-bearing component of cell, increases in thickness. It accounts for 0.7–0.8% of cell’s dry weight in exponential phase cells whereas in stationary phase it increases up to 1.4–1.9% (Mengin-Lecreulx and van Heijenoort, 1985). At the subcellular level, nucleoid condensation occurs for DNA protection, the cytoplasm gets condensed with an overall decrease in protein synthesis as a consequence of stress or stationary phase (Navarro Llorens et al., 2010). At the translational level, the 70S ribosomes are converted into inactive 100S ribosome dimers by associating with ribosome modulation factor (Wada, 1998). This process, termed as ribosome hibernation, is thought to be a mechanism to fine-tune the translation process according to environmental conditions (McKay and Portnoy, 2015). Recently, 16S rRNA fragmentation at the tip of helix 6 has been shown to attenuate the activity of 30S ribosomal subunit and thereby protein synthesis (Luidalepp et al., 2016). Also, during limited nutrient availability, accumulation of truncated mRNA and deacylated tRNA occurs. The ribosomes become stuck on these mRNAs and owing to the absence of a stop codon, the ribosome is unable to get released (Pletnev et al., 2015). These mechanisms are understood to be the defense response upon starvation. As a result of the various morphological, metabolic, transcriptional, or translational alterations, the stationary phase cells become resistant to high temperature, high concentrations of H_2_O_2_, and very high medium osmolarity.

**FIGURE 2:**
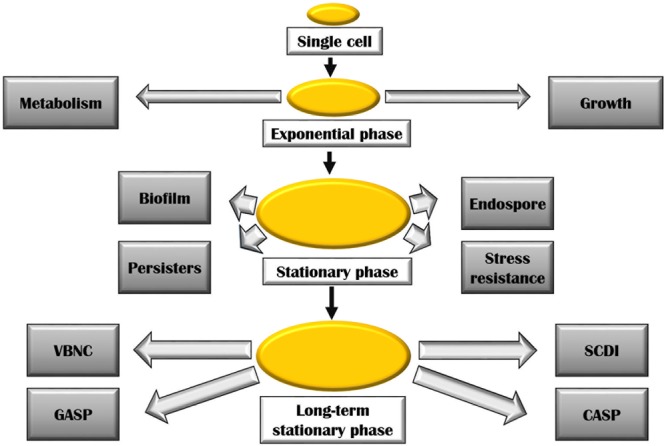
Various bacterial adaptations at stationary and long-term stationary phase. Abbreviations are described in the text.

Cells in exponential, stationary, and long-term stationary phases have different fates (**Figure 2**). As a consequence of starvation, many bacteria including the genera *Bacillus* and *Clostridium* form resistant spores helping them withstand the harsh surrounding environment. Non-optimal growth conditions also lead to the formation of biofilm in many bacterial species. Physiologically, biofilm bacteria are similar to stationary phase bacteria. One key transition is the formation of persisters induced during stationary phase, in biofilms, and also as a consequence of a general stress response. These cells could also arise in exponential growth by the activation of ppGpp as a consequence of sub-lethal antibiotic concentration. The formation of these bacterial persisters is understood to be the reason behind relapsing infections and is a major cause of drug resistance (Harms et al., 2016).

During the late stationary phase sometimes referred to as long-term stationary phase, several remarkable adaptations take place. On continued starvation, one of the survival strategies includes bacteria entering a viable but non-culturable state (VBNC). In this state, bacteria remain metabolically active but fail to form colonies on bacteriological media. Several bacteria including *Rhodococcus biphenylivorans* (Su et al., 2015), *Escherichia coli, Agrobacterium tumefaciens, Helicobacter pylori, Lactococcus lactis*, many *Vibrio* species, and *Pseudomonas* species have been shown to enter the VBNC state (Oliver, 2005). The VBNC state poses a serious health risk as the dormant bacterial species could remain undetected in culturable conditions, though having the ability to cause infections (Navarro Llorens et al., 2010). A variety of stresses is said to lead to the manifestation of VBNC state (Pletnev et al., 2015). Prolonged starvation also results in Growth Advantage in Stationary Phase (GASP) phenotype. The GASP phenomenon is a result of mutations in the *rpoS* allele (described later) which confers a gainful ability to continue growing during starvation conditions, thus replacing the parental population (Navarro Llorens et al., 2010). These mutations allow the mutants to effectively scavenge the nutrients released by dead cells (Zambrano and Kolter, 1996). A number of Gram-positive bacteria such as *Listeria monocytogenes* (Bruno and Freitag, 2011), *Staphylococcus aureus, Enterococcus faecalis*, and *Bacillus globigii* (Finkel et al., 1997) and Gram-negative bacteria including *Campylobacter, Geobacter, Vibrio, E. coli, Pseudomonas*, etc., have been found to enter the GASP state (Chen and Chen, 2014). Gefen et al. (2014) coined the term ‘constant activity stationary phase’ (CASP) to describe the phenomenon of constant rate of protein synthesis observed in non-growing bacteria that have undergone over more than 60 h of starvation. On studying the protein production at this stage, they have found that both the protein synthesis machinery including ribosomes, RNA polymerases, etc., and resources such as amino acids, nucleotides, etc., remain constant at CASP. Finally, constant promoter activity was observed in this experiment for up to 10 h of starvation. Another interesting phenomenon experienced by bacterial population in stationary phase is the ‘stationary phase contact-dependent inhibition’ (SCDI). It requires physical contact between the evolved and original bacteria (Lemonnier et al., 2008). In this process, it was observed that the evolved strains either killed or inhibited the growth of bacteria that they were derived from. The inhibiting ability of these strains is attributed to mutations within a single gene involved in glycogen synthesis pathway: *glgC* (encoding ADP-glucose pyrophosphorylase). Astonishingly, all evolved strains overproduced glycogen which seemed to be necessary for SCDI to occur (Navarro Llorens et al., 2010).

## Alternative Sigma Factors Active At Stationary Phase

A key regulator of stationary phase gene expression in *E. coli* is the transcription factor σ^S^ [a product of *rpoS* (*katF*) gene]. The *E. coli* genome was found to contain two genes *katE* and *katG* encoding for HPII and HP1w1-4x catalases. The expression of HPII was highest in stationary phase and has been shown to be completely dependent on *katF* gene product. The latter serves as sigma factor for RNA polymerase and therefore named as *rpoS* or σ^S^ or σ^38^ or stationary phase sigma factor or starvation sigma factor (Tanaka et al., 1997).

The amount of σ^S^ remains relatively low in the growing phase of cells but increases markedly when the cell encounters stress, starvation or enters stationary phase. The role of this protein is to aid in survival and improved resistance to stressful conditions. Induction of σ^S^ is observed under conditions of low pH, heat or cold shock, UV-induced DNA damage, nutrient starvation, high cell density, high osmolarity, etc. (Hengge, 2011). The σ^S^-dependent genes have been attributed to morphological changes (Hengge, 2011), induction of starvation proteins (Alexander and St. John, 1994), iron uptake, carbohydrate metabolism, amino acid transport, and so on, at the onset of stationary phase (Lacour and Landini, 2004).

The *rpoS* sigma factor is selectively utilized in stationary phase. The major sigma factor rpoD (σ^70^) is inhibited by a regulator of sigma D (Rsd). The rationale for σ^S^ selectivity *in vivo* is not completely understood, but it is known that many promoters can exhibit both σ^S^ and σ^70^ mediated expression *in vitro.* It is well known that σ^70^ is affected by changes in spacer region and consensus –10 and –35 positions, but the alternative σ^S^ is shown to be less affected by changes in these regions, thus making it more selective *in vivo* (Hengge, 2011). Another observation by Tanaka et al., 1995 indicates that the –35 region is not always required for stationary-phase expression (Tanaka et al., 1995). In this study, the *fic* promoter was shown to function with promoter sequences downstream from –17. Also, the promoters recognized by RpoS are found to contain curved DNA region. Hence, the absence of consensus –35 and the presence of curved DNA region imparted σ^S^ dependence to galP1 and galP2 promoters, whereas the presence of –35 sequence in the same promoter changed the specificity toward σ^70^ (Kolb et al., 1995). Thus, the general belief is that the σ^S^ promoters lack a –35 consensus sequence. However, some authors have suggested CTGCAA (Bohannon et al., 1991) or CCGACA (Wise et al., 1996) as the –35 consensus sequence. Similarly for –10, Hengge-Aronis (1993) has suggested a consensus sequence of TATACT, which was later changed to CTATACT (Espinosa-Urgel et al., 1996). More recently, a long consensus sequence KCTAYRCTTAA for –10 region has been proposed, where K could be T or G, Y could be T or C, and R could be A or G (Weber et al., 2005). Not all the stationary-phase induced genes depend on σ^S^, and out of the many genes that show higher level of expression in the stationary phase, only 10% is known to be dependent on σ^S^ (Rava et al., 1999). Out of the genes induced in stationary phase, those that show σ^S^ independent behavior are *dnaK, groEL, htpG* which depend on σ^32^ (Kolter et al., 1993).

Several other alternative sigma factors have been reported. In *Salmonella typhimurium*, σ^E^ has been suggested to serve a complementary role in stationary phase survival. Mutants deficient in *rpoH* gene coding for σ^E^ have been shown to be susceptible to oxidative stress (Testerman et al., 2002).

The number of sigma factors varies from 1 in *Mycoplasma genitalium* (Dorman, 2011), 6 in *Gordonia* sp. IITR100 (Jaishankar et al., 2017), 7 in *E. coli* (Ishihama, 1997), 18 in *B. subtilis* (Gruber and Gross, 2003), 24 in *Pseudomonas aeruginosa* (Potvin et al., 2008), and 65 in *Streptomyces coelicolor* (Kim et al., 2008). **Table 1** gives a list of various sigma factors in well-known bacterial species and the types of sigma factors upregulated at stationary phase.

**Table 1:** List of sigma factors upregulated at stationary phase in different bacteria.

Name of the organism	Sigma factors	Sigma factors upregulated at stationary phase	Reference
*Escherichia coli*	**7** σ^70^(σ^D^), σ^24^(σ^E^), σ^28^(σ^F^), σ^32^(σ^H^), σ^54^(σ^N^), σ^38^(σ^S^), σ^18^(σ^Fecl^)	σ^32^(σ^H^), σ^54^(σ^N^), σ^38^(σ^S^)	Ishihama, 1997
*Bacillus subtilis*	**18** σ^A^, σ^B^, σ^C^, σ^D^, σ^E^, σ^F^, σ^G^, σ^H^, σ^K^, σ^L^, σ^M^, σ^V^, σ^W^, σ^Y^, σ^X^, σ^Z^, Xpf, YlacC,	σ^B^, σ^C^, σ^D^, σ^H^	Haldenwang, 1995; Gruber and Gross, 2003
*Streptomyces coelicolor*	**65**	σ^B^, σ^F^, σ^H^, σ^M^, σ^N^, σ^R^	Kim et al., 2008; Tripathi et al., 2014
*Pseudomonas aeruginosa*	**24**	σ^E^, σ^H^, σ^S^	Potvin et al., 2008
*Corynebacterium glutamicum*	**7** σ^A^, σ^B^, σ^C^, σ^D^, σ^E^, σ^H^, σ^M^	σ^B^, σ^H^, σ^M^	Pátek and Nešvera, 2013

## Regulation of RpoS

The RpoS is regulated at post-transcriptional level by *rpoS* mRNA secondary structure, small RNAs, Hfq, and HU proteins, ClpXP protease and RssB (phosphorylation-modulated RpoS recognition factor) (Hengge-Aronis, 2002). The *rpoS* mRNA is stimulated by regulatory factors such as Hfq (HF-1) protein and DsrA (small regulatory RNA) and repressed by H-NS (histone-like protein) and *oxyS* RNA. The 5′ UTR of *rpoS* mRNA forms a loop which represses its translation. This loop can be disrupted by non-coding RNAs such as *dsrA, rprA*, and *arcA* (Gaida et al., 2013). Another sRNA which positively regulates *rpoS* mRNA is *gcvB* (Jin et al., 2009).

The turnover of RpoS protein in exponential phase is very high with a half-life of 1.4 min (Lange and Hengge-Aronis, 1994). The RpoS protein is stable in stationary phase.

The levels of RpoS are also controlled by a number of other factors. These include both positive regulators such as ppGpp and polyphosphate (polyp) and negative regulators such as cAMP and UDP glucose.

The availability of ppGpp is dependent on RelA, a ppGpp synthase that is associated with ribosomes. In stationary phase, when the uncharged tRNAs accumulate due to decreased availability of amino acids, *relA* is turned on and synthesizes ppGpp. This turns on the promoters involved in amino acid biosynthesis and uptake (Barker et al., 2001). It has been shown that 6S RNA regulates *relA* gene expression, which leads to alteration in ppGpp levels in stationary phase (Cavanagh et al., 2010). The rRNA genes are turned off by ppGpp. Many stationary phase promoters (SPPs) are also regulated by 6S RNA, even in the absence of ppGpp.

In *B. subtilis*, it has been demonstrated that cells entering in stationary phase have small GTP and GDP pools. This is possibly due to conversion of GTP to (p)ppGpp or due to the lack of sufficient precursors available for nucleotide synthesis. Lopez and coworkers demonstrated that treatment of cells with decoyinine, an inhibitor of GMP synthase, can result in induction of stationary phase genes (Ratnayake-Lecamwasam et al., 2001).

The intracellular levels of certain compounds such as trehalose, glycine betaine, glycogen, and polyphosphate are high under stress conditions. Some of these compounds modulate function of the RpoS holoenzyme. For example, glutamate and trehalose modulate the holoenzyme binding to promoters. Similarly, altered promoter selectivity has been observed in *E. coli* when RpoS associates with inorganic polyphosphate. The inhibition due to PolyP is relieved by high concentrations of potassium glutamate (Shimada et al., 2004). Bacterial pheromone, Homoserine lactone (HSL), a small molecule responsible for communication between bacteria, also affects the concentration of σ^S^ in the cell. Mutants in the biosynthetic pathway for synthesis of HSL loose the ability to induce σ^S^ (Zambrano and Kolter, 1996).

## Expression of Genes in Stationary Phase

When the cells are growing, the metabolism-linked genes are highly expressed, and get turned off when the cells enter stationary phase. The stationary phase is a period of no growth, however, genes essential for survival of organisms are expressed at this stage. Around 20% of the genes of *E. coli* are found to express at higher level in the stationary phase (Rava et al., 1999). These genes are directly linked to many key events including DNA repair, glycogen production, thermotolerance, osmotolerance, etc. (Bohannon et al., 1991; Ishihama, 1997). Transcriptome profiling/expression analysis of *E. coli* in stationary phase revealed upregulation of genes which are involved in survival during osmotic stress (*ots, tre, osm*), long-term survival (e.g., *bolA, dps, cbpA*, and *glgS*), periplasmic shock (*rpoE* and *rseA*), cold shock (*csp* genes), etc. Other genes include carbon storage regulator (*csrA*), trp repressor binding protein (*wrbA*) and universal stress protein (*uspA*) (Chang et al., 2002). Moreover, several antibiotics including lactocin B of lactic acid bacteria, alfatoxin of *Aspergillus* species are produced mainly in stationary phase (Matin, 1992).

**FIGURE 3:**
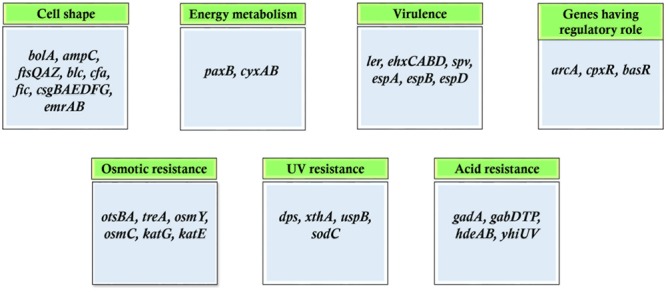
Categories of genes transcribed in stationary phase.

Persister cell formation has also been attributed to genes differentially expressed in stationary phase. These cells are recalcitrant to antibiotic treatments and often are the major cause of drug resistance. Several polyamines including putrescine, spermidine, and cadaverine direct persister formation through upregulation of genes such as *rpoS, rmf, yqjD* (Tkachenko et al., 2017). This observation suggests that polyamine metabolism participates in the regulation of persister cells formation. To determine the genes upregulated at stationary phase microarray was done in *Mycobacterium smegmatis* grown under conditions of glycerol and glucose depletion. Different subset of genes were identified that were preferentially upregulated at stationary phase. The categories of genes included those involved in metabolism of sulfur, sigma factors including *sigB, sigE*, and *sigH*, fatty acid degradation, anaerobic respiration, etc. (Hampshire et al., 2004). Also, of key interest in this study is the presence of stationary phase operons involving many gene clusters that were significantly upregulated in stationary phase. On investigating further, the presence of other such operons were also found. The *pdh* operon of *Streptococcus mutans* is expressed only in the stationary phase. This operon was observed to be transcribed only by a subpopulation of bacteria in stationary phase and was vital for survival during long periods of sugar starvation. The *pdh* operon consists of four genes that are transcribed as an operon: *pdhD, pdhA, pdhB, pdhC*, which encode the components of PDH (pyruvate dehydrogenase) complex, i.e., pyruvate dehydrogenase (two subunits encoded by *pdhA* and *pdhB*), dihydrolipoyl transacetylase (*pdhC*), and dihydrolipoyl dehydrogenase (*pdhD*). The inactivation of the first gene: *pdhD* resulted in impaired survival in both batch cultures and biofilms (Busuioc et al., 2010). Similarly, phage shock protein operon (*pspABCE*) of *E. coli* was reported to be critical for survival under prolonged stationary phase at alkaline conditions. This operon was expressed strongly under extreme stressful conditions and remained significant for survival under nutrient-limited conditions (Weiner and Model, 1994). Categories of genes that are preferentially upregulated in stationary phase is shown in **Figure 3**. Studies have demonstrated that starved cells exhibit more protective resistance to different stresses as compared to resistance induced during growing stage by non-lethal exposure of stresses (Kolter et al., 1993).

## Stationary Phase Promoters

The genes expressed in stationary phase are controlled by promoters, which result in induction of stationary phase. The promoters, which are turned on, are called SPPs. They are recognized by RNA polymerase holoenzyme containing σ^S^ and therefore called RpoS.

The vast importance of SPPs had been realized way back in 1980s with the study of *mcbA* promoter and *bolA* P1 promoter of *E. coli* (Connell et al., 1987; Aldea et al., 1989). The *mcbA* promoter causes increased level of transcription initiation for Microcin B17, a DNA replication inhibitor. Promoter *mcbA*-LacZ fusion showed the induction of transcription in nitrogen, phosphate, and carbon starvation conditions. Similarly, *bolA-lacZ* fusion demonstrated an increase in expression of approximately 10- to 20-fold during transition to stationary phase. Since then many SPPs have been isolated and characterized in both Gram-positive and Gram-negative bacteria (**Tables 2, 3**). Particularly regarding *E. coli* and *B. subtilis*, the stationary-phase-specific gene regulation has been intensively studied (Hengge, 2011).

**Table 2:** Stationary phase promoters in Gram-negative bacteria.

Name of or	Name of promoter	Gene product	–10	–35	Other motifs	Length of spacer	Reference
*Escherichia coli*	*BolAp1*	BolA	CGGCTAGTA	CTGCAA	–	15	Aldea et al., 1989
	*TreA*	TreA (Osmotically inducible periplasmic trehalase)	ATGCAG	TAAGGT	–	17	Repoila and Gutierrez, 1991
	*Cst*-1	Cst	–	–	–	–	Tunner et al., 1992
	*Fic*	Fic (PABA or folate)	TATACT	–	–	–	Utsumi et al., 1993
	*Hns*	H-NS	TATTAT	TTGCAC	–	17	Dersch et al., 1993
	*PoxB*	PoxB (pyruvate oxidase)	TAAACT	–	–25: CGTCA; –60: GTTAGTG	–	Chang et al., 1994
	*Slp*	Slp	TATTATG	GATGAAA	–	16	Alexander and St. John, 1994
	*AldB*	AldB (Aldehyde dehydrogenase)	TACCCT	–	–	–	Xu and Johnson, 1995
	*CsiD*	CsiD (Carbon starvation inducible gene)	TATTTT	TGCGCA	–	17	Marschall et al., 1998
	*OsmY* (*Csi*-5)	OsmY (Periplasmic protein of unknown function)	TATATT	CGAGCG	–	15	Lange et al., 1993; Becker and Hengge-Aronis, 2004
*Shigella flexneri*	*GadA*	GadA (Glutamate decarboxylase)	CTACTTT	–	–	–	Waterman and Small, 2003
*Vibrio anguillarum*	*EmpA*	EmpA mettaloprotease	GATCCA	CCGTGCTAC		19	Croxatto et al., 2004; Denkin and Nelson, 2004

**Table 3:** Stationary phase promoters from Gram-positive bacteria.

Organism	Promoter	Gene product	–10	–35	Length of spacer (bp)	Reference
*Bacillus subtilis*	*Pst*	Phosphate-specific transport	TTTACT	TTCAAA	18	Qi et al., 1997
*Bacillus subtilis*	*Cry3a*	Crystal proteins	TAAGCT	TTGCAA	18	Lee et al., 2010
*Bacillus subtilis*	*Ylb*	–	TACAAT	TTGGA	18	Yu et al., 2015
*Bacillus subtilis*	*SrfA* mutant	Srf operon (lipopeptide antibiotic surfactin)	TTGACT	TATAAT	–	Guan et al., 2016
*Streptomyces coelicolor*	*KasO* mutant	Colemycin P1	TAAAGT	TTGACA	18	Wang et al., 2013
*Corynebacterium glutamicum*	*Cg3141* mutant	*Cg3141 (flavohemoprotein)*	TGGGAT	TTAAGG	17	Kim et al., 2016
*Gordonia sp.* IITR100	Stationary phase promoter	–	AATAAT	TTAACT	22	Singh et al., 2016

**FIGURE 4:**
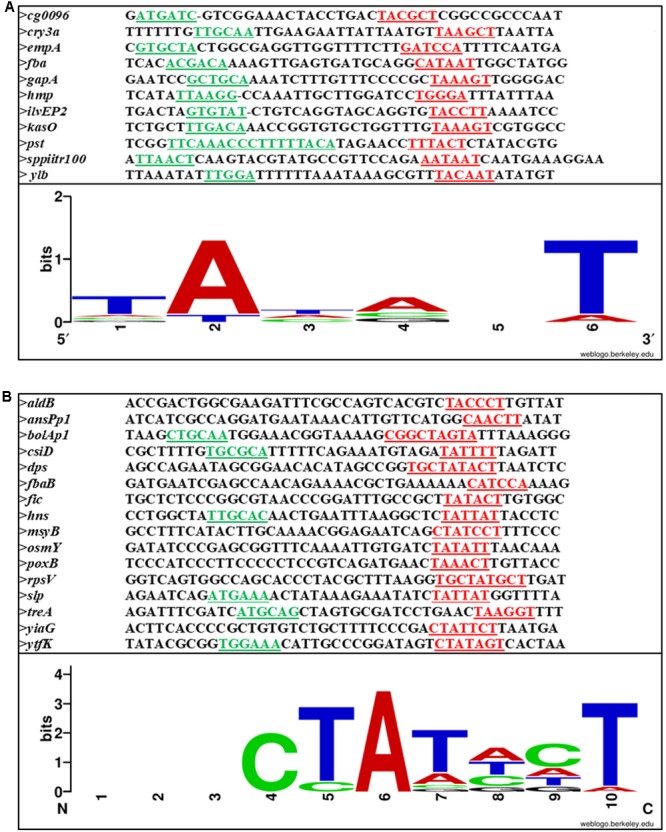
Sequence alignment of **(A)** Gram-positive and **(B)** Gram-negative stationary phase promoters. –10 and –35 are underlined and shown in red and green, respectively. The conserved bases are shown below.

On analysis of the different SPPs, our observation is that there is not much variation between this class of promoters and σ^70^ promoters. It is the sequence outside the –10 and –35 regions that distinguish between σ^70^- and σ^S^-dependent promoters. **Figures 4A,B** shows the –10, –35 and spacer region of few SPPs from Gram-positive and Gram-negative bacteria and the consensus sequence at the –10 region is shown as a logo designed using WebLogo software available online (Crooks et al., 2004).

Among the SPPs exist a special class of promoters known as the gearbox promoters which include *mcbAp, bolAp1, ftsQp* to name a few. This class of promoters has been studied in several Gram-negative bacteria including *E. coli*. Two different highly conserved consensus –10 and –35 sequence have been proposed by Aldea et al. (1993) for this class of promoters: CTGCAA or GTTAAGC at –35 position and CGGCAAGTA or CGTCC at –10 position. Gearbox promoter-induced gene expression seems to correlate inversely with growth rate and these promoters may or may not depend on σ^S^.

## Energy Reserves Consumed During Stationary Phase and Source of Nutrients for Protein Production

During unfavorable conditions of growth, reprogramming the cellular machinery for sustaining viability is a natural process of adaptation. Reserve polymers like glycogen and poly-β-hydroxybutyric acid that are accumulated by bacteria during growth are rapidly consumed during conditions of carbon starvation to ensure survival. In case of bacteria that do not accumulate these polymers, cellular RNA is rapidly degraded for energy generation (Matin, 1992). Among RNA, rRNA is preferentially degraded (Deutscher, 2003). Besides, 50% of ribosomes synthesized during exponential growth are degraded during entry to stationary phase (Piir et al., 2011). What is surprising is that, when in stationary phase, these ribosomes are fairly stable and so degradation occurs only in between the stages.

The yield of protein production from stationary phase systems is as high as 121% as compared to their log phase counterparts (Ou et al., 2004). This raises a very important question: What makes protein synthesis possible at stationary phase?

Balaban and coworkers devised a microfluidic device and followed the production of fluorescent proteins at stationary phase. They found that cells after entering stationary phase continue to produce proteins for several days (Gefen et al., 2014). It has been suggested that cells continue to produce proteins at stationary phase by reusing amino acids from degraded proteins. Moreover, the biosynthetic pathway of a few amino acids including serine, aspartate/asparagine, glutamine/glutamate, and alanine were shown to be active during stationary phase (Shaikh et al., 2010). In addition, it is shown that each condition resulting in starvation results in induction of specific set of proteins (Kolter et al., 1993).

## Development of Gene Expression Systems Using Stationary Phase Promoters

A strong promoter is the key for developing efficient gene expression systems. For recombinant protein production, several bacterial hosts have been used as cell factories, with features such as easy purification, improved protein folding and secretion, high production of membrane proteins, etc. (Ferrer-Miralles and Villaverde, 2013). To develop more such expression systems in bacteria, it is necessary to ensure proper selection of a promoter that would drive the expression of genes at the right time and with maximum amount.

Promoters could be classified as constitutive or inducible, growth-stage limited, tissue specific, etc. Inducible promoters can further be classified into inducer-specific and auto-inducible promoters. Constitutive promoters are not useful for toxic proteins. Inducer-specific promoters involve the cost of inducer. Also, some chemical inducers such as Isopropyl-β-D-1-thiogalactopyranoside (IPTG) are expensive and toxic (Cao and Xian, 2011). Further, the addition of external inducers often requires growth monitoring which is vital for productivity and hence lead to difficulty in fermentation.

Auto-inducible promoters are ideal for large-scale protein production as they are induced at late log phase or stationary phase. Such promoters induce expression of the recombinant gene without any inducer and thus are economical. However, most of them have low activity (Yu et al., 2015). In *B. subtilis*, Fan and coworkers successfully identified a strong SPP Pylb by microarray approach (Yu et al., 2015). The β-galactosidase activities were observed to be up to 5000 miller units. The authors have proposed that such a promoter will be useful for protein production. A SPP-based auto-inducible gene expression system has been constructed using cry3Aa promoter. The Pcry drives the expression of crystal proteins in *B. thuringiensis*. The promoter cry3Aa was tested in *B. subtilis* and the wild type have the LacZ levels up to 1000 miller units and on mutagenesis resulted in levels up to 5200 miller units (Lee et al., 2010). Similarly, in another Gram-positive bacteria, *Gordonia* sp. IITR100, a SPP was identified and the β-galactosidase activities were up to 600 miller units (Singh et al., 2016). However, the β-galactosidase activities vary with respect to strain, copy number of plasmid, growth medium, temperature, etc., so it is difficult to assess the strength of promoter based on Miller units alone. In future, a study of such promoters based on the number of transcripts would be useful to compare the strength.

In *Corynebacterium glutamicum*, promoter of *cg3141* gene coding for flavohemoprotein was found to show higher inducibility in the stationary phase. Then, a synthetic promoter library was prepared to change the spacer and flanking regions in the promoter, to obtain a range of promoter strengths (Kim et al., 2016). At the end, one of the synthetic promoters that showed up to 20-fold higher strength compared to the original *cg3141* promoter was obtained and demonstrated for fed-batch cultivation of glutathione S-transferase in a 5L reactor. **Table 4** depicts the list of SPP-based expression vectors constructed till date. Studies like these indicate that the potential of SPPs is phenomenal. In *Streptomyces*, a high-level recombinant protein expression system has been patented (US Patent No. 7,316,914).

**Table 4:** Stationary phase promoter–based gene expression systems reported from Gram-negative and Gram-positive bacteria.

Vector	Promoter	Organism	Ori	Antibiotic resistance	Reporter gene	Reference
pTGV1	*BolAp*	*E. coli*	pBR322	Ampicillin	*bolA*-*LacZ*	Aldea et al., 1989
pFL1	*Fic*	*E. coli*	pMB1	Tetracycline	*LacZ*	Utsumi et al., 1993
pKS4, pKS5	*Hns*	*E. coli*	pSC101	Tetracycline	*Hns*-*LacZ*	Dersch et al., 1993
pNH5	*OsmY (Csi5)*	*E. coli*	pBR322	Ampicillin	*Csi5*-*LacZ*	Lange et al., 1993
pMC719	*Slp*	*E. coli*	pBR322	Tetracycline	*Slp*-*LacZ*	Alexander and St. John, 1994
pYYC128	*PoxB*	*E. coli*	pBR322	Chloramphenicol	*PoxB*-*LacZ*	Chang et al., 1994
pRJ4025	*AldB*	*E. coli*	pBR322	Ampicillin	*LacZ*	Xu and Johnson, 1995
pYQ23	*Pst*	*Bacillus subtilis*	pBR322	Ampicillin, Chloramphenicol	*LacZ*	Qi et al., 1997
pCM3	*CsiD*	*E. coli*	pMB1	Ampicillin	*Csi-LacZ*	Marschall et al., 1998
pDM35-EmpA	*EmpA*	*Vibrio anguillarum*	R6K	Chloramphenicol	*LacZ*	Croxatto et al., 2004; Denkin and Nelson, 2004
pGRP (many promoters)	Many	*E. coli*	pBR322	Ampicillin	green fluorescent protein (*eGFP*)	Shimada et al., 2004
pD82-aprE	*Cry3a*	*Bacillus subtilis*	–	Chloramphenicol	*AprE-LacZ*	Lee et al., 2010
pMD-ficD	*Fic*	*E. coli*	pBR322	Ampicillin	*phlD*	Cao and Xian, 2011
pDR4-K^∗^	*KasO*	*Streptomyces coelicolor*	EBV origin	Hygromycin	*XylE*-*neo*	Wang et al., 2013
pBSG03	*SrfA*	*Bacillus subtilis*	pBR322	Ampicillin, Kanamycin, Neomycin	*fp*	Guan et al., 2015
Pylb-bgaB-pUBC19	*Ylb*	*Bacillus subtilis*	pUBC19	Ampicillin	Beta-gal (*bgaB*)	Yu et al., 2015
pSC1	Stationary phase promoter	*Gordonia sp.* IITR100	pRC4	Kanamycin	*LacZ*	Singh et al., 2016
pCES-P_4_-N_14_-sfGFP	P_4_-N_14_	*Corynebacterium glutamicum*	pCG1	Kanamycin	*sfGFP*	Kim et al., 2016

## Applications

**FIGURE 5:**
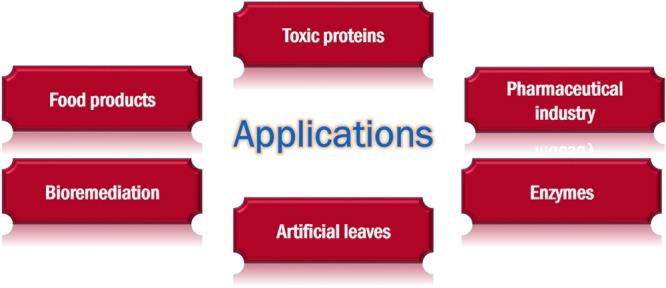
Applications of stationary phase gene expression systems.

SPPs have immense potential for use in many industries (**Figure 5**).

Recombinant production of toxins whose overproduction is detrimental to the growth of cells needs controlled conditions for expression. In such cases, the use of SPP is advantageous as the overproduction will not affect the growth of the host cells. Many bacteria have been used to demonstrate the utility of cell-density-dependent expression systems for heterologous protein production. Metabolic engineering of bacteria for enhanced production of industrially important chemicals has been carried out since a long time. The *fic* promoter of *E. coli* was used to express *phlD* gene at a higher titer in stationary phase, without the addition of any inducer, for the production of phloroglucinol, which has utility in pharmaceutical industry and plant tissue culture. After 20 h of cultivation in a flask with shaking, 9% of glucose supplied had converted to phloroglucinol with a productivity of 0.014g/l h (Cao and Xian, 2011). *B. subtilis* has been engineered for overproduction of aminopeptidase using a mutated P*srfA* system and has resulted in 87.89 U/ml of enzyme activity (Guan et al., 2015). Using *B. subtilis*, a *cry*-promoter-based system was developed wherein cellulose and alkaline protease were produced with a higher yield as compared to the wild-type cry3A promoter (Lee et al., 2010).

It is a well-known fact that the non-growing phase of lactic acid bacteria accounts for a major proportion of flavor production in lactic acid bacteria (van de Bunt et al., 2014). Therefore, engineering bacterial cells in such a way that they are expressed at high levels, during the ripening process, by using SPPs would enhance their applicability in food industry.

In the bioremediation industry, microorganisms have routinely been employed for removing pollutants. Due to low nutrient availability in polluted sites, genetic engineering of cells resulting in higher enzymatic activities at lower growth rates have been shown to be highly efficient for bioremediation process. On studying the phenol degradation capability of two non-growing recombinant *E. coli* strains, it was found that the *groEL-*promoter-driven gene expression system caused 75% phenol degradation while the *tac*-promoter-driven expression could cause only 15% degradation of phenol (Matin, 1992). As suggested by Tunner et al. (1992), it is possible to use starvation-induced promoters for chemical waste biodegradation wherein enzymes can be induced naturally by bacteria due to the occurrence of nutrient-limited conditions in the environment. This could save the cost of induction thereby increasing the efficiency of the process.

In a very interesting experiment, *Rhodospirillum rubrum* cells grown photoheterotrophically, evolved hydrogen for about 70 h after growth ceased (Melnicki et al., 2008). Similarly, a purple non-sulfur photosynthetic bacterium, *Rhodopseudomonas palustris* under nitrogen starvation conditions, produced hydrogen gas for over 4000 h thus paving way for creation of ‘artificial leaves’ (Gosse et al., 2010).

## Conclusion and Future Prospects

Stationary phase survival is a means of bacterial adaptation by which bacteria survive under conditions of stress or starvation. The ugly aspect of this is that such a mechanism results in the persistence of pathogenic bacteria which can cause relapsing of infections. However, the good side is represented by the various biotechnological applications that have come up recently based on the promoters of the genes which are upregulated at stationary phase. In the present review, we have discussed not only the changes at the cellular and molecular levels at stationary phase, but also the various promoters characterized, their regulation and the gene expression systems developed. There are still many unknowns. For example, very little is known about the proteins which are involved in chromosome organization and their interaction with DNA at stationary phase. Such proteins could be important players in regulating gene expression at stationary phase. Further very few SPPs have been experimentally characterized till date. Such promoters should be highly useful for protein production as the growth and protein production phase can be uncoupled. This will pave way toward constructing improved gene expression systems for recombinant protein production.

## Author Contributions

JJ and PS wrote and edited the manuscript.

## Conflict of Interest Statement

The authors declare that the research was conducted in the absence of any commercial or financial relationships that could be construed as a potential conflict of interest.
